# Development and Characterization of New Species Cross-Reactive Anti-Sialoadhesin Monoclonal Antibodies

**DOI:** 10.3390/antib5020007

**Published:** 2016-03-23

**Authors:** Marjorie De Schryver, Hanne Van Gorp, Inge Hoebeke, Bauke De Maeyer, Karen Ooms, Isabel Pintelon, Louis J. Maes, Paul Cos, Hans J. Nauwynck, Peter L. Delputte

**Affiliations:** 1 Laboratory of Microbiology, Parasitology and Hygiene, University of Antwerp (UA), Antwerp 2610, Belgium; marjorie.deschryver@uantwerpen.be (M.D.S.); louis.maes@uantwerpen.be (L.J.M.); paul.cos@uantwerpen.be (P.C.); 2 Laboratory of Virology, Ghent University, Merelbeke 9820, Belgium; hanne.vangorp@irc.vib-ugent.be (H.V.G.); ingehoebeke@hotmail.com (I.H.); baukedemaeyer@gmail.com (B.D.M.); karen.ooms@ugent.be (K.O.); hans.nauwynck@ugent.be (H.J.N.); 3 Inflammation Research Center, VIB-Department of Internal Medicine, Ghent University, Ghent 9000, Belgium; 4 Laboratory of Cell Biology and Histology, University of Antwerp (UA), Antwerp 2020, Belgium; isabel.pintelon@uantwerpen.be

**Keywords:** sialoadhesin, monoclonal antibody, internalization

## Abstract

Sialoadhesin (Sn) is a surface receptor expressed on a subset of macrophages in steady state conditions. During inflammation and diseases, Sn is highly upregulated on macrophages and blood monocytes. Therefore, therapies using monoclonal antibodies (mAbs) to target Sn-positive (Sn^+^) cells are a potential strategy for targeted treatment. It has been shown that Sn internalizes after binding with a mAb, though it is not clear whether this is species-specific. In this study, new Sn-specific mAbs were developed and analyzed for cross-reactivity between species. In addition, the newly developed mAbs were compared to mAbs used in previous research for their epitope recognition and other Sn-specific characteristics. Both species-specific and cross-reactive antibodies could be identified. Furthermore, sialic acid-binding of red blood cells (RBC) could be inhibited with mAbs recognizing different epitopes and all mAb showed internalization of Sn. The newly developed mAbs can be used as novel tools for Sn research and further analysis of Sn internalization in different species.

## 1. Introduction

Sialoadhesin (Sn), also known as Siglec-1 or CD169, is a member of the family of sialic acid-binding immunoglobulin-like lectins (Siglecs) and is exclusively expressed on a subset of resident tissue macrophages in steady state conditions [1,2,3]. Sn is the largest Siglec, containing 16 constant domains and an N-terminal variable domain that is able to recognize sialic acids [4]. Although Sn is conserved between different species, the amino acid sequence of Sn can vary substantially. Interestingly, the cytoplasmic tail is poorly conserved in length and amino acid sequence, with only 30% of the amino acid sequence being identical between human and mouse [3,5,6,7]. Furthermore, Sn does not contain any known signalization motif in the cytoplasmic tail, which is in contrast with most other Siglecs [4,5,7].

Sn, as a member of the Siglec family, can bind specifically with sialic acid ligands found on cell surfaces [8,9]. Therefore, Sn was hypothesized to function as a cell–cell receptor. Sn can indeed bind with different cells of the granulocytic lineage in a sialic acid-dependent manner, like bone marrow cells, neutrophils, and blood leukocytes [4,10,11]. Besides cells of the granulocytic lineage, Sn is able to bind with red blood cells (RBC) in a sialic acid-dependent manner, resulting in the formation of a rosette structure when these RBC bind to Sn-expressing macrophages. This was the first identified property of Sn, as reflected in the initial name for Sn being sheep erythrocyte receptor (SER) [12]. The binding of RBC to Sn can be blocked by monoclonal antibodies (mAbs) close to the sialic acid-binding domain of Sn [4,13,14].

Although the exact role of Sn is still unknown, different characteristics of Sn were identified and gave a better understanding of the potential role of Sn and Sn-positive (Sn^+^) macrophages. Sn can be drastically upregulated on macrophages, monocytes, and monocyte-derived dendritic cells (DC) during conditions of inflammation [1,2,3,13,15,16]. It was shown that Sn^+^-macrophages can act as antigen (Ag) presenting cells after targeting Sn with mAbs cross-linked with Ag. This leads to Ag presentation and the activation of B-, T-, and invariant natural killer T-cells [16,17,18,19,20]. Some bacteria, protozoa, and viruses containing sialic acids on their surface can interact with Sn^+^-macrophages [21,22]. Bacteria *Neisseria meningitidis* and *Campylobacter jejuni* and protozoa *Trypanosoma cruzi* interact with Sn in a sialic acid-dependent manner and aid the phagocytosis of the pathogen [23,24,25]. Porcine reproductive and respiratory syndrome virus (PRRSV) and human immunodeficiency virus type 1 (HIV-1) contain sialic acids on their envelope and are able to interact with Sn^+^-macrophages and Sn^+^-DC respectively [6,26,27,28]. Interestingly, for all these pathogens, the binding and uptake in the cell can be inhibited or reduced after addition of anti-Sn mAbs [14,23,24,25,29]. After the discovery of sialic acid-dependent internalization of pathogens through Sn, internalization was further studied. Liposomal nanoparticles were produced with sialic acid on their surface to target Sn and deliver their cargo, which results in the activation of invariant natural killer T-cells [16,30,31].

Antibody-induced internalization was previously studied for other Siglecs. Anti-CD33 and anti-CD22 mAbs were also shown to induce internalization and targeting of these Siglecs is a therapeutic approach to target malignant cells [32,33]. Similar to other Siglecs, internalization of Sn after mAb triggering was studied and internalization was observed for porcine (pSn), human (hSn), and murine Sn (mSn) [6,30,34]. MAb-induced internalization of pSn occurs in a clathrin-dependent manner, after which antibodies are located in early endosomes [35]. The mAb-induced internalization of pSn was documented by different research groups using two different mAbs, 41D3 and 1F1, but no direct comparison was done. Both mAbs are able to inhibit RBC binding, indicating that they both recognize a region close to the sialic acid-binding domain [36,37]. However, it is not clear if these mAbs recognize the same epitope. Previous research of other surface proteins showed that internalization is different for mAbs recognizing a different epitope [38,39]. Also for Sn, it might be possible that the epitope recognition of the mAb is important for the internalization of Sn. Previous studies analyzed the importance of the cytoplasmic tail of Sn. Sn-mutants without a cytoplasmic tail resulted in a decreased infection of virus that utilizes Sn as an internalization receptor, which can be due to a lower expression of the Sn-mutant or a loss of internalization capacity [40]. Similarly, adding a di-aromatic motif in the cytoplasmic tail of Sn resulted in internalization of HIV-1, while binding of HIV-1 to the wild-type Sn only results in invagination of the Sn–HIV-1 complex [34]. Yet, such motif has not been identified and since the cytoplasmic tail of Sn is poorly conserved between species, a different mechanism of mAb-induced internalization might be possible. This is preferentially analyzed using cross-reactive mAbs, which are not available yet, to rule out antibody-specific effects [7,34,40].

In this study, we developed new anti-Sn mAbs to obtain a broader range of available anti-Sn mAbs, and possibly species cross-reactive mAbs. In addition, we aimed to further investigate the internalization of mSn, where sometimes conflicting results are described regarding its capacity to internalize [30,41]. We compared the newly developed anti-Sn mAbs with anti-Sn mAbs already described in previous research. For hSn, the most commonly used mAb is 7D2, produced by immunizing mice with a soluble and truncated form of hSn that only contains the first four N-terminal domains of hSn [3]. MAb 3D6 is an anti-mSn mAb, produced by immunizing rats with purified mSn [10]. MAb 41D3, an anti-pSn mAb, was developed by immunizing mice with porcine alveolar macrophages [42]. Most research regarding the internalization of Sn was performed with these mAbs, however contradicting results were observed by using mAb 3D6 and 7D2 to induce mSn or hSn internalization, respectively [30,41]. In this study, all experiments were performed in Chinese hamster ovary cells (CHO) cells expressing a recombinant Sn (Sn^+^-CHO) to overcome differences that might arise from the use of primary cells expressing Sn and the different species origin of the cells. We also evaluated the cross-reactivity of the mAbs and analyzed their functionality using immunofluorescence staining and Western blot analysis, and the mAbs were clustered according to the epitopes they recognize. Finally the capacity of the mAbs to inhibit the sialic acid-dependent binding and the capacity to induce internalization of Sn was evaluated.

## 2. Results and Discussion

### 2.1. Development and Characterization of mAbs

A first immunization was done in separate rats using two different conserved peptides from the 6th extracellular domain of hSn, pSn, and mSn. This resulted in 17 different hybridomas that were screened using peptide-based and cell-based enzyme-linked immunosorbent assay (ELISA). All the mAbs were able to recognize the conserved peptides used for initial immunization. However, none of them reacted with Sn^+^-CHO cells, suggesting that these linear epitopes were not accessible in the native protein (data not shown).

Since this first approach was not successful, a second immunization was set up in which, respectively, mice were immunized with cells expressing hSn and rats with the extracellular domain of mSn fused to a murine IgG2a Fc backbone. Obtained anti-hSn hybridomas were screened by ELISA for recognition of hSn, while for the anti-mSn hybridomas, a negative selection was first performed to eliminate hybridomas that recognize the murine IgG2a Fc backbone, followed by a positive selection for the recognition of mSn. This resulted in a selection of 14 hybridoma clones recognizing recombinant hSn and two hybridoma clones recognizing recombinant mSn. Moreover, ELISA results showed that nine of these anti-hSn hybridoma cross-reacted with pSn but no cross-reactivity was observed with the mSn clones (Table 1). All mAbs had an IgG1 kappa isotype, except 26B2 and 17B12, which had an IgG2b kappa isotype (Table 1).

**Figure 1:**
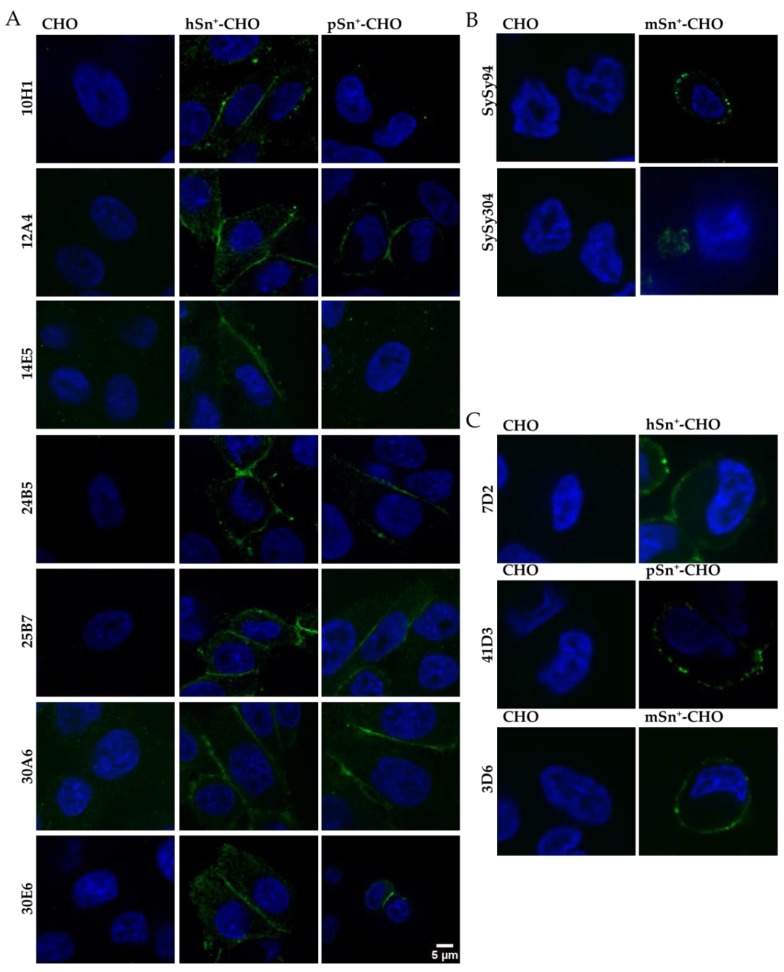
Microscopic analysis of surface staining of sialoadhesin (Sn) on CHO cells. Monoclonal antibodies (mAbs) were added to fixed Sn^+^ CHO cells to analyze the recognition of surface Sn. Secondary Alexa Fluor 488-labeled antibodies were added to bind with the mAbs (green), nuclei were stained with 4′,6-diamidino-2-phenylindole (DAPI) (blue). Wild-type CHO cells were used as control cells. (**A**) All anti-hSn mAbs were tested against hSn^+^-CHO and pSn^+^-CHO; (**B**) anti-mSn mAbs were used to stain mSn^+^-CHO; (**C**) reference mAbs 7D2, 3D6, and 41D3 were analyzed for recognition of hSn^+^-, pSn^+^-, and mSn^+^-CHO cells, respectively.

Further screening of the different mAbs was performed using microscopic analysis and flow cytometry. For immunofluorescent staining, mAbs were added to Sn^+^-CHO cells and wild-type CHO cells were used as the negative control. The newly developed mAbs were compared to the reference mAbs 7D2, 3D6 and 41D3, which were previously developed by other groups and have been extensively used [3,10,42]. Based on immunofluorescent staining, only seven of the 14 ELISA-positive clones resulted in a staining of hSn^+^-CHO cells and gave no background staining on wild type CHO cells. Only five of these mAbs (12A4, 24B5, 25B7, 30A6, and 30E6) also stained pSn, yet with a lower intensity (Figure 1A). For the anti-mSn mAbs only mAb SySy94 resulted in a clear staining of mSn^+^-CHO cells. MAb SySy304 also stained mSn^+^-CHO cells, yet the staining was less clear and limited to the bottom of the cell that makes contact with the plate (Figure 1B). All reference mAbs gave a clear surface staining (Figure 1C).

**Figure 2:**
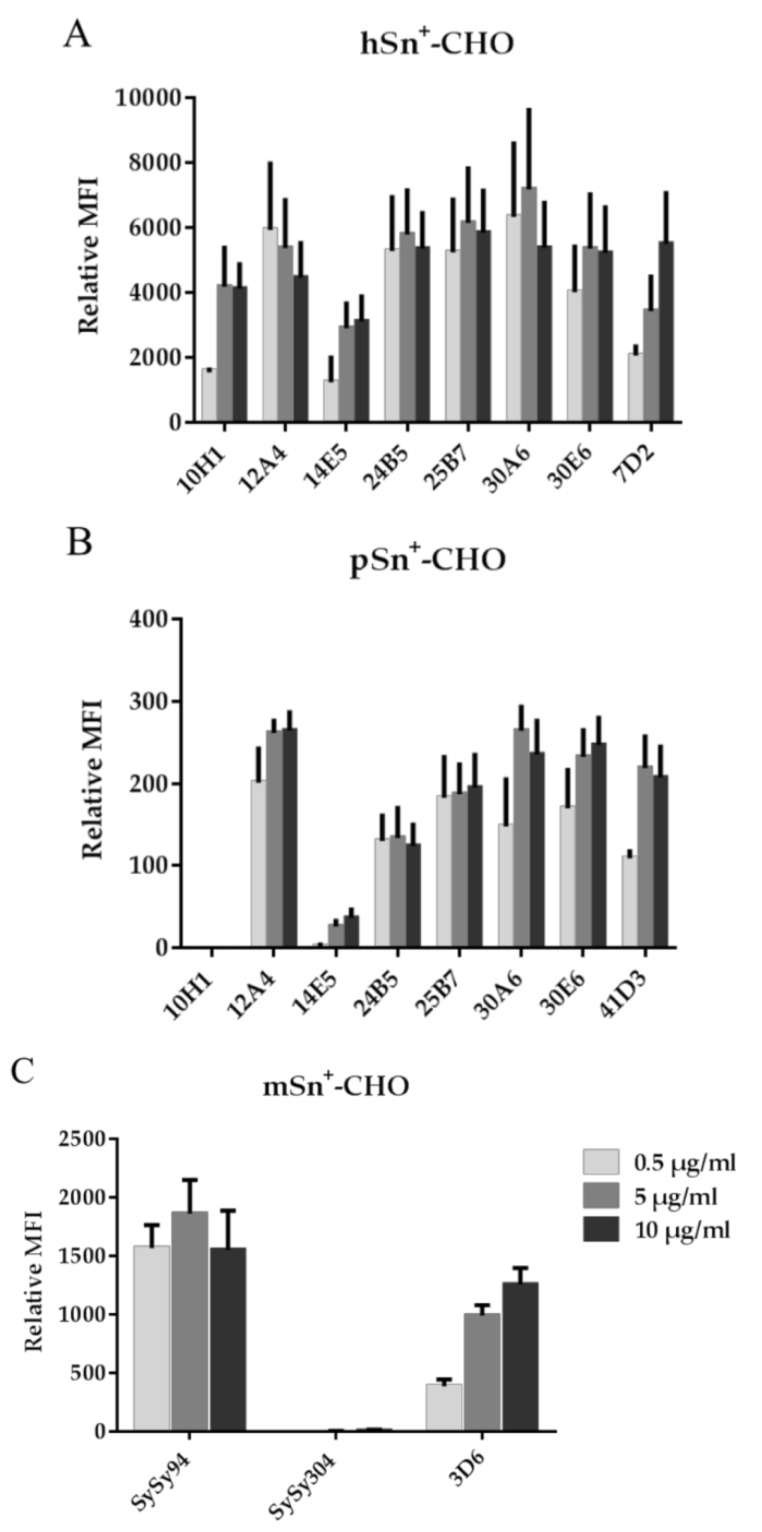
Flow cytometric analysis of mAbs binding to Sn^+^-CHO cells. Different concentrations of mAbs were added to CHO cells expressing (**A**) hSn; (**B**) pSn; and (**C**) mSn, followed by secondary fluorescently-labeled Ab. Surface staining was measured only on living cells by excluding dead cells using propidium iodide (PI). Data represents the relative mean fluorescence intensity (MFI) and standard error of the mean of three independent repeats.

The capacity of the mAbs to recognize Sn was also analyzed with flow cytometry. Different concentrations of mAbs were added to the cells to give an indication of the relative binding capacity of the newly developed mAbs compared to the reference mAbs. All seven anti-hSn mAbs were able to recognize cell surface-expressed hSn. Staining with mAbs 12A4, 24B5, 25B7, and 30A6 resulted in a higher fluorescent signal at lower mAb-concentrations compared to the reference mAb 7D2. Only mAb 14E5 had a lower fluorescent signal relative to all other anti-hSn mAbs (Figure 2A). For pSn, the same five mAbs that recognized pSn in previous experiments also recognized pSn using flow cytometry. MAbs 12A4, 30A6, and 30E6 gave a stronger signal at a lower mAb concentration than the reference mAb 41D3 using pSn^+^-CHO cells as a target; however, this difference was not significant. MAbs 10H1 and 14E5, only recognizing hSn in previous experiments, resulted in no or very low fluorescent signals when staining pSn^+^-CHO cells (Figure 2B). For murine Sn, only mAb SySy94 recognized mSn, mAb SySy304 gave no staining even at the highest mAb concentration (Figure 2C). Several of the new mAbs were able to recognize Sn at a lower mAb concentration, suggesting a higher relative binding capacity compared to the reference mAb, especially for mAb SySy94, which is significantly different compared to mAb 3D6. Based on these data, a selection of mAbs was made for further experiments, which is indicated in Table 1 with an asterisk.

### 2.2. MAb Recognition of Sn on Western Blot

**Figure 3:**
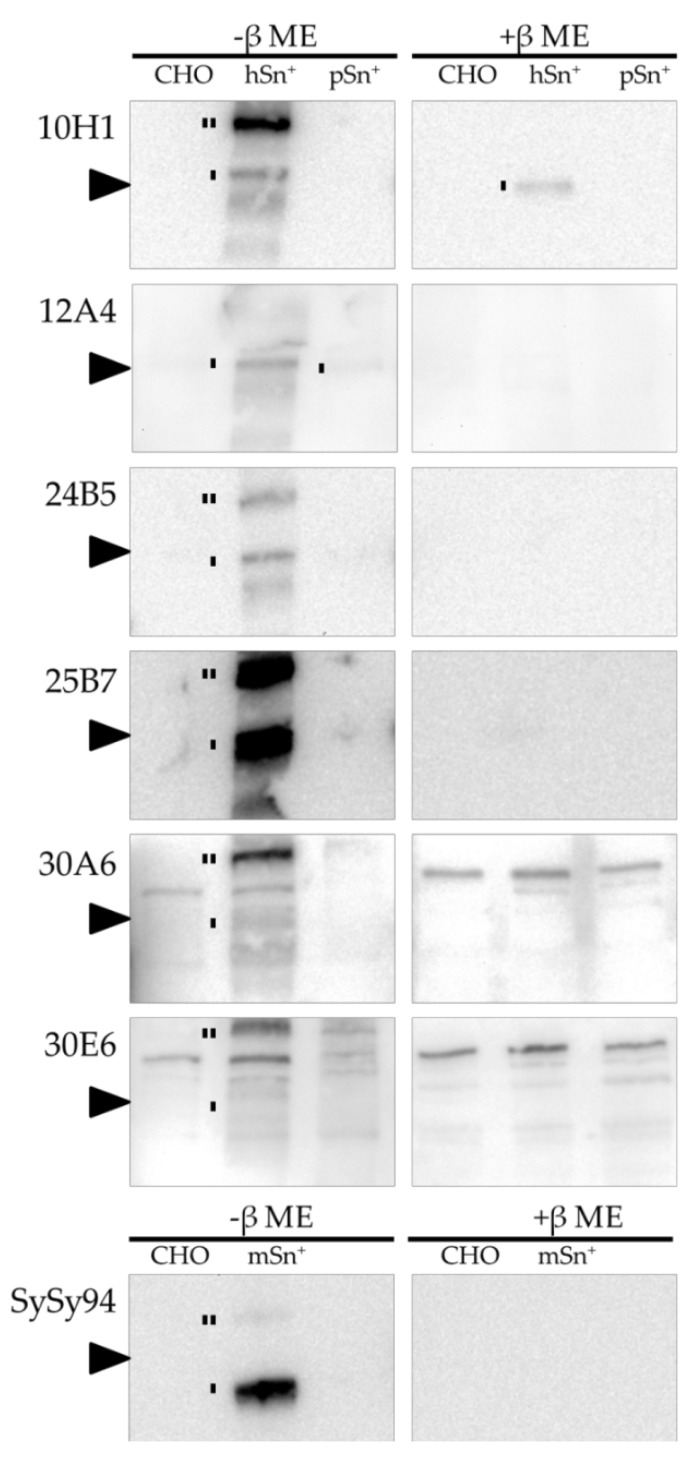
Western blot analysis of the different mAbs. Lysates of CHO, hSn^+^-CHO, pSn^+^-CHO, and mSn^+^-CHO were run under non-reducing (-β mercaptoethanol (ME)) and reducing (+β ME) conditions. The newly developed mAbs were added to the blots followed by an anti-mouse or anti-rat horseradish peroxidase (HRP)-labeled secondary antibody. Sn monomers and dimers are indicated with one line and two lines respectively. The arrow indicates 220 kDa.

Anti-Sn reference mAbs were used in previous research to recognize Sn on Western blot. All mAbs 7D2, 3D6, and 41D3 were previously shown to recognize Sn in both reducing and non-reducing conditions [3,6,43]. The newly developed mAbs were analyzed for their ability to recognize Sn on Western blot under reducing and non-reducing conditions. CHO cells with and without Sn were lysed and the Western blot membranes were stained with the different newly developed mAbs, as shown in Figure 3. Wild-type CHO cells were used as negative control where no Sn staining was visible in both reducing and non-reducing conditions. HSn was detected only in non-reducing conditions by all newly developed mAbs except for mAb 10H1, which also recognized Sn in reducing conditions. For mAbs 10H1, 24B5, 30A6, and 30E6, a dimer of Sn was also observed. MAbs 30A6 and 30E6 were the only newly developed mAbs with less recognition of hSn in Western blot analysis. In addition, non-specific signal was also observed using these mAb, indicating that these mAbs are not suitable for Western blotting. Interestingly, none of the cross-reactive mAbs were able to recognize pSn using Western blot analysis, except for mAb 12A4 with a low intensity. MAb SySy94 was able to recognize mSn only in non-reducing conditions, as a monomer and dimer. These results show that all mAbs, except for mAb 10H1, recognize an epitope of Sn only found in non-reducing conditions and not in reducing conditions. For further research using Western blot techniques, mAb SySy94 can be used to detect mSn and mAb 25B7 is the best mAb for hSn recognition but not for pSn.

### 2.3. Clustering of the Anti-Sn mAb Based on a Competitive Binding Assay

Clustering of the different mAbs based on a mAb-competition assay was previously shown for other proteins using a combination of biotinylated and non-biotinylated mAbs [44,45]. An excess of unbiotinylated mAbs was added to the cells and were allowed to bind to surface-expressed Sn for 60 min at 37 °C. Afterwards, biotinylated-mAbs were added and their capacity to bind was evaluated with flow cytometry using fluorescein isothiocyanate (FITC)-labeled streptavidin. If the epitopes recognized by both mAbs are the same or in close proximity so that steric hindrance may occur, biotinylated-mAbs will not bind to Sn or will do so less effectively. In this case, no fluorescent signal or a lower one will be detected. However, if the biotinylated-mAbs interact with another epitope, a clear fluorescent signal will be detected. As a positive control, the same mAb is used both unbiotinylated and biotinylated and the low fluorescence signal detected in this setting is used as the reference signal that indicates that the mAbs bind the same epitope or are in very close proximity.

**Figure 4:**
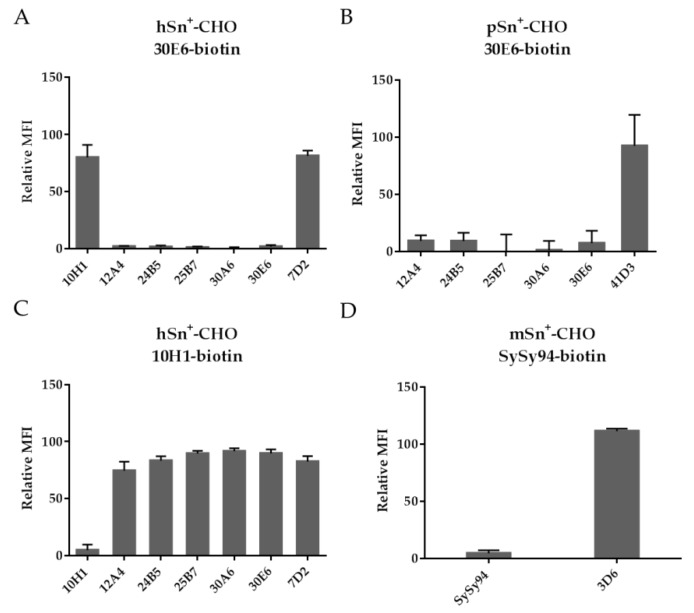
Competitive binding assay to cluster mAb binding using flow cytometry. An excess of mAbs was added to Sn^+^-cells. After 60 min of incubation, biotinylated-mAbs were added and unbound mAbs were removed after 60 min. Fluorescently-labeled streptavidin was added to stain only the bound biotinylated-mAbs. Dead cells were excluded using PI. A high fluorescent signal indicated the mAb had a different epitope than the biotinylated-mAb. MAb 30E6 was biotinylated and added to (**A**) hSn^+^-CHO and (**B**) pSn^+^-CHO. Biotinylated mAbs 10H1 and SySy94 were added to (**C**) hSn^+^-CHO and (**D**) mSn^+^-CHO, respectively. The relative MFI was determined comparing the fluorescent signal of the cells to that of cells with no competition taking autofluorescence of the cells into account. The relative MFI and standard error of the mean was determined for three independent repeats.

MAb 30E6 was chosen as the first mAb to be biotinylated since it recognizes both hSn and pSn. These experiments (Figure 4A,B) suggest that all mAbs recognizing both hSn and pSn (12A4, 24B5, 25B7, 30A6, and 30E6) bind the same epitope on Sn. However, the hSn-specific mAbs 10H1 and 7D2, and the pSn-specific mAb 41D3, recognized another epitope (*p* < 0.05). Based on these results, mAb 10H1 was biotinylated to compare the epitope with mAb 7D2. All mAbs had a high fluorescent signal except for mAb 10H1, suggesting that mAb 10H1 recognizes a different epitope compared to the other newly developed anti-hSn mAbs and the reference mAb 7D2 (*p* < 0.05) (Figure 4C). Finally, mAb SySy94 was biotinylated to compare the epitope with mAb 3D6. The fluorescent signals showed that the epitope for mAbs SySy94 and 3D6 is significantly different (Figure 4D). An overview of the clustering of the different mAbs is given in Table 2.

### 2.4. Capacity of mAbs to Block the Sialic Acid-Binding of Sn

One of the first discovered properties of Sn is the ability to interact with sialic acids on red blood cell (RBC) resulting in the formation of rosettes, a feature that is conserved in all species studied [4,13,14]. The binding of RBC to Sn can be blocked using mAbs but only if the mAb binds to or is in close proximity to the sialic acid-binding domain of Sn, which is located in the N-terminal variable domain [13,46]. The reference mAbs 7D2, 3D6, and 41D3 were all able to interfere with RBC binding, as documented in previous research, suggesting that they all bind to or are in close proximity to the sialic acid-binding domain of Sn [3,10,14,42]. Yet, it has also been shown that mAbs can interfere with binding of RBC to Sn even when recognizing different epitopes; this was illustrated for mAbs SER-4 and 3D6, both recognizing mSn [10]. In this study, different concentrations of mAbs were added to Sn^+^-cells to allow binding with Sn before adding human RBC. Unbound RBC were removed and rosette formation was analyzed using light microscopy. A cell with at least four bound RBC was considered a positive cell [14].

**Figure 5:**
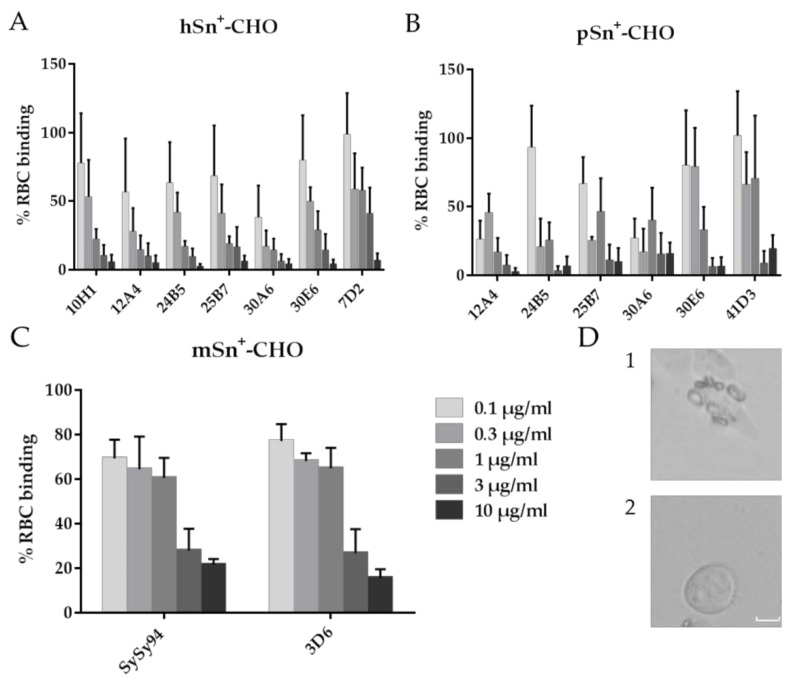
Analysis of red blood cell (RBC) binding inhibition on Sn^+^-cells. (**A**) hSn^+^-; (**B**) pSn^+^-; and (**C**) mSn^+^-CHO cells were treated with *Vibrio cholerae* neuraminidase to remove sialic acids on the cell surface before adding different concentrations of mAbs. MAbs were able to bind Sn and prevent RBC binding before RBC were added. Unbound RBC were washed away and rosette formation was analyzed with light microscopy. Cells were counted to obtain a percentage of positive cells, which are cells bound to at least four RBC. Data show the results of three independent repeats with standard error of the mean. (**D**) Representative light microscopic images of RBC binding to Sn^+^-cells in the (1) absence or (2) presence of an anti-Sn mAb that blocks sialic acid binding. Scale bar: 10 µm.

All anti-hSn mAbs were able to inhibit the RBC binding on hSn^+^-cells in a dose-dependent manner (Figure 5A). The highest concentration used almost entirely inhibited RBC binding to hSn. Our results suggest that a higher concentration of mAb 7D2 is needed for the same inhibition of the RBC binding compared to the newly developed mAbs. MAb 30A6 seemed to inhibit the RBC binding better compared to the other anti-hSn mAbs, since the inhibition of this binding already occurred at low mAb concentrations (Figure 5A). For pSn, all mAbs could inhibit RBC binding to pSn in a dose-dependent manner. MAbs 12A4 and 30A6 inhibited the RBC binding already at low concentrations compared to the other mAbs but with no significant difference (Figure 5B). Anti-mSn mAbs SySy94 and 3D6 reacted similarly to the inhibition of RBC binding in a dose-dependent manner (Figure 5C). To conclude, these results suggest that the newly developed, different mAbs recognize different epitopes and could all bind to the sialic acid-binding domain of Sn or in close vicinity. For research where blocking of the sialic acid-binding domain is important, such as preventing binding of sialylated pathogens, mAb 30A6 could be used for both hSn and pSn at already low concentrations.

### 2.5. Capacity of mAbs to Induce Internalization of hSn, pSn, and mSn

Although Sn was originally described as a receptor only involved in cell–cell interactions, Sn is capable of internalization after binding of specific ligands, such as anti-Sn mAb. This feature was first observed with pSn, which internalizes upon binding of mAb 41D3 [6]. Another mAb was also shown to induce internalization of pSn, however no direct comparison was made between these mAbs [6,18,35]. Similar to pSn, hSn and mSn were also shown to internalize after mAb triggering; however, some contradicting results were described [26,30,34,40,41]. Therefore, the newly developed mAbs were evaluated for their capacity to induce Sn internalization and compared to the reference mAbs.

**Figure 6:**
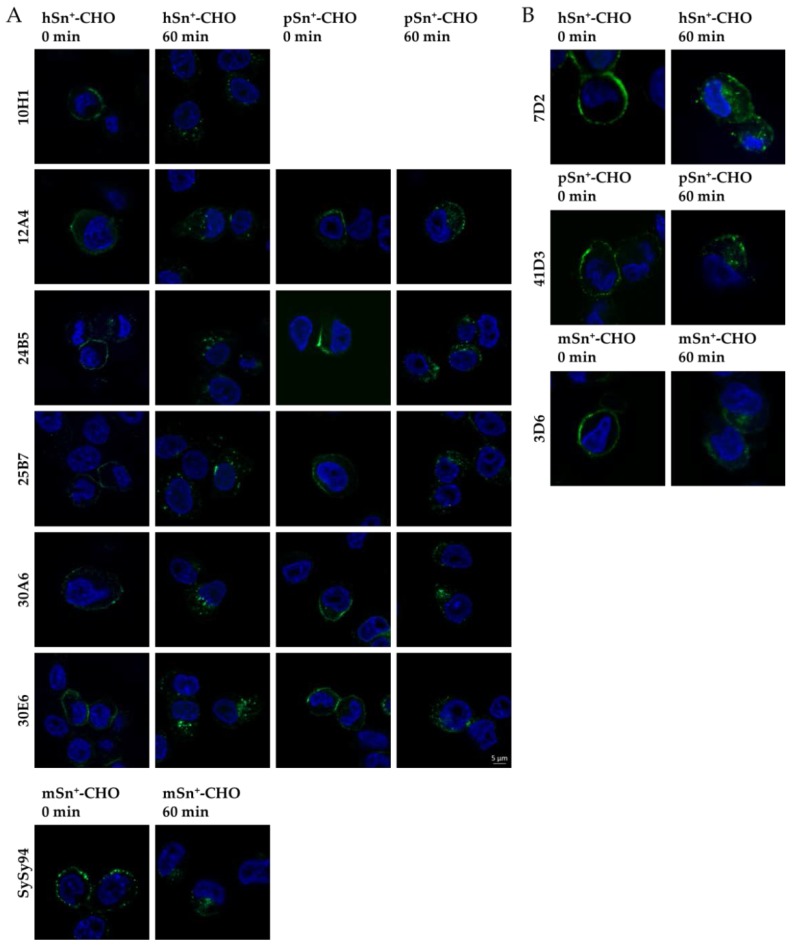
Internalization of Sn induced by different Sn-specific mAbs. (**A**) Newly developed mAbs and (**B**) reference mAbs were added to the cells for 60 min at 4 °C to allow only surface staining. Cells where then fixed (0 min) or were brought to 37 °C to induce internalization (60 min). Cells were permeabilized so internalized vesicles could be observed. Secondary Alexa Fluor 488-labeled antibodies were added to the cells (green) and DAPI was used to stain the nuclei (blue).

Internalization of Sn was studied by immunofluorescent stainings in combination with confocal microscopy and flow cytometry. Using confocal fluorescence microscopy, a clear surface staining was visible at time 0 for all mAbs. When internalization was allowed for 60 min, intracellular vesicles were observed for hSn, pSn, and mSn using all different mAbs (Figure 6; Figure S1). These results show that the newly developed mAbs and reference mAbs were able to induce internalization after binding with Sn. Although some contradicting results have been described for mSn internalization using mAb 3D6, our results indicate that mAb 3D6 is able to induce internalization of mSn.

**Figure 7:**
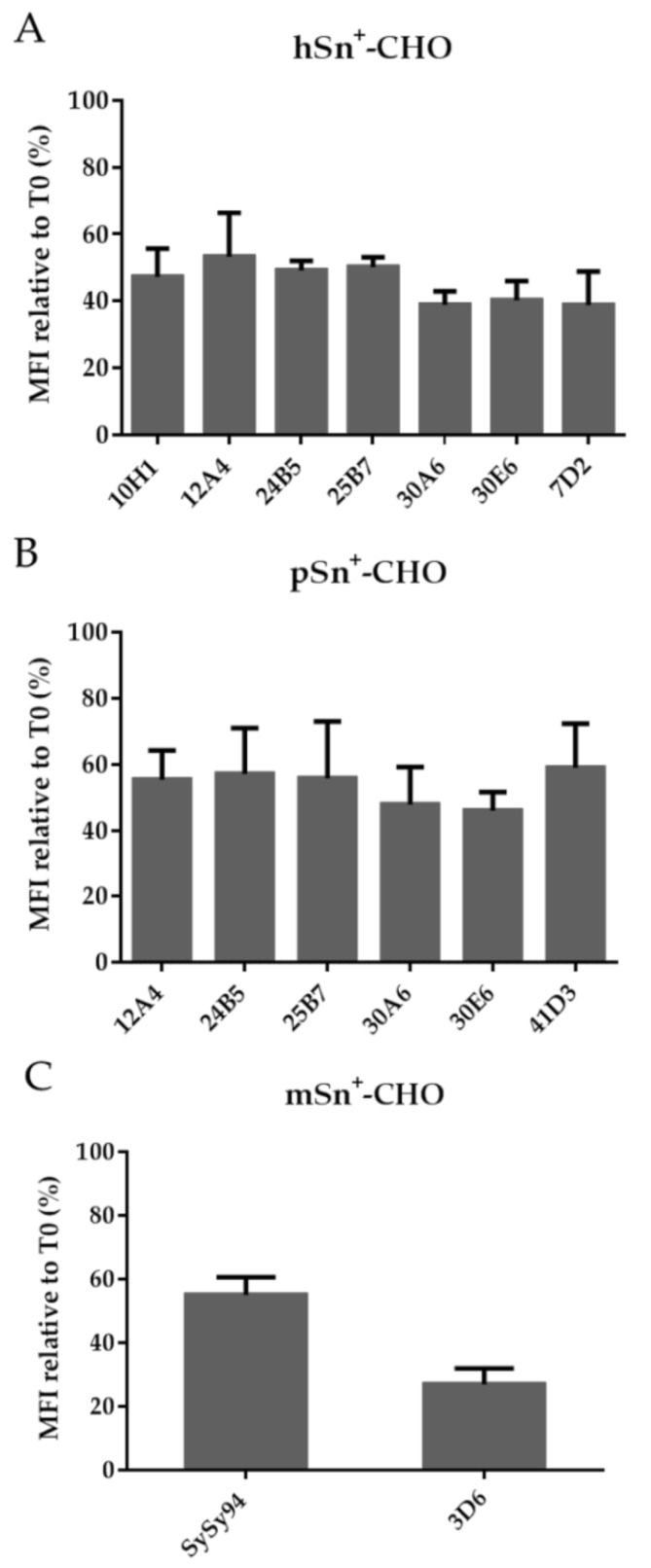
Flow cytometric analysis of internalization capacity of different mAbs on Sn^+^-CHO cells. The cells were incubated with different mAbs to allow only binding to surface-expressed (**A**) hSn; (**B**) pSn; and (**C**) mSn. Afterwards the cells were shifted to 37 °C during 60 min to induce internalization of Sn. Secondary fluorescently-labeled antibodies were added to the cells, staining only remaining surface-Sn. Only viable cells were analyzed with flow cytometry using PI staining. Data represent the mean and standard error of the mean of three independent repeats. Relative internalization was calculated by comparing the fluorescent signal to that of cells without internalization.

Internalization of Sn was also analyzed with flow cytometry. MAbs were added to the cells at 4 °C to allow surface staining before internalization was induced by shifting the cells to 37 °C. Fluorescently-labeled antibodies were added to non-permeabilized cells to stain only cell surface-expressed Sn. Upon internalization, less fluorescent signal was observed. Furthermore, the results showed that not all surface Sn is internalized into the cells. This was also previously illustrated for mAbs 7D2 and 41D3 resulting, respectively, in 68% and 60% Sn remaining on the surface [34,35]. All mAbs induced internalization and approximately 50% and 60% of hSn and pSn, respectively, remained on the cell surface and no significant differences were observed between mAbs tested. Interestingly, a small difference in internalization was observed for the cross-reactive mAbs less hSn remained on the surface in contrast to pSn (Figure 7A,B). This suggests that, although the mAbs are cross-reactive, binding to hSn might be more efficient. For mSn, 3D6 induced a higher reduction of Sn surface expression compared to SySy94 (*p* < 0.05) (Figure 7C). This difference can be explained by the difference in relative binding capacity as previously observed for these mAbs. Another explanation could be that mSn has multiple conformations on the cell surface that are differentially recognized by Sn-specific mAbs, and that do not internalize with the same efficiency after mAb binding. Also, not all Sn^+^-cells show internalization upon mAb binding, as previously shown [35]. In addition, binding of the mAb to different epitopes could also result in different characteristics of Sn-internalization. Such differences have been described for other surface receptors using mAb that recognize different epitopes of the same receptor [38,39]. Clearly, more research is needed to analyze the mAb-induced internalization of Sn in more detail.

## 3. Materials and Methods

### 3.1. Ethical Statement

The animal experiments were evaluated and authorized by the Ethical Committee of the Faculty of Veterinary Medicine of Ghent University (Permit number EC2010/161).

### 3.2. Production of hybridoma cells and screening

Alignment of the amino acid sequence of Sn from different species revealed two peptides that are conserved in hSn, pSn, and mSn, but not in rat Sn (_520_AEVVEGQAVTLSCRS_534_ and _559_SSLLLPAASSTDAGSY_574_). These peptides were synthesized, linked to the immune carrier keyhole limpet hemocyanin (KLH), and used for immunization of rats. The developed hybridomas were screened by ELISA for the recognition of the peptides and surface-expressed Sn.

An antibody against hSn was made by immunizing mice with Mouse Embryonic Fibroblast cells that were transduced with full-length hSn using a retrovirus system. The hybridomas were screened by ELISA of their supernatant against recombinant hSn with a C-terminal His-tag (R&D Systems, Oxon, UK) and CHO-K1 cells stably expressing hSn.

Antibodies against mSn were raised by immunization of rats with recombinant mSn with C-terminal murine IgG2a (R&D Systems). The hybridoma supernatants were screened by ELISA against recombinant murine Sn and murine IgG2a to exclude hybridomas that produce antibodies against the Fc-part of recombinant mSn, which has an IgG2a isotype.

MAbs were produced and purified by culturing the hybridoma cell lines in standard Falcon T-flasks. To scale up the production, Triple Falcons were used. The serum used in the culture medium was first depleted of the bovine immunoglobulins by running it on a protein G affinity column. In some experiments, serum was replaced by Insulin-Transferrin-Selenium-Ethanolamine supplement (ITS–X; Life Technologies, Grand Island, NY, USA). Purification of the mAbs from the hybridoma supernatant was performed on a protein G affinity column using a peristaltic pump.

Isotyping of the different anti-hSn and anti-mSn mAbs was performed using a mouse Immunoglobulin Isotyping ELISA Kit and rat isotyping kit from BD Biosciences respectively.

Produced mAb were compared to reference mAb, 7D2 (Thermo Scientific, Rockford, IL, USA), 3D6.112 (Antibodies online, Aachen, Germany) and 41D3 [42].

### 3.3. Cells

CHO-K1 cells, kindly provided by Dr. J. D. Esko (University of California, San Diego, CA, USA), were transfected with pSn to obtain a stable cell line [47], or transduced using a retroviral system. These cells were grown in F-12K medium supplemented with 10% heat inactivated fetal bovine serum (Life Technologies).

### 3.4. Recognition of Sn and internalization assay

Cells were seeded on a coverslip for 2 h. Primary mAbs, were added to the cells during 60 min and cells were kept on ice to allow surface staining. Afterwards cells were directly fixed with 4% paraformaldehyde (PF; Merck, Molsheim, France) or first shifted to 37 °C to stimulate internalization and then fixed. Cells were permeabilized using Triton X-100 (Sigma-Aldrich, Schnelldorf, Germany) and Alexa Fluor 488-chicken anti-mouse or Alexa Fluor 488-chicken anti-rat (Life Technologies) secondary antibodies were added to label the primary antibody. Nuclei were stained with DAPI (Sigma-Aldrich). Confocal images were obtained using Apotome 2 with an Axio Observer inverted microscope and a Compact Light Source HXP 120C with Filter set 49 and 10 for blue and green fluorophores respectively (Zeiss) and using a Nikon Eclipse Ti-E inverted microscope attached to a microlens-enhanced dual spinning disk confocal system (UltraVIEW VoX; PerkinElmer, Zaventem, Belgium) equipped with 405 and 488 nm diode lasers for excitation of blue and green respectively. Images were processed using ImageJ software (New York, NY, USA).

### 3.5. Flow cytometric analysis

Cells in suspension were kept on ice during the entire experiment in a 96-well plate with V-shaped bottom. Antibodies were added in different concentrations to the cells and mAbs were allowed to bind with surface Sn during 60 min at 4 °C. Unbound mAbs were washed away and internalization was induced by shifting the cells to 37 °C. Internalization was stopped by cooling the cells to 4 °C. For further processing, cells were kept on ice to prevent internalization. Secondary antibodies, Alexa Fluor 488-chicken anti-mouse for pSn or Alexa Fluor 647-goat anti-mouse (Life Technologies, Frederick, MD, USA) for hSn and Alexa Fluor 488-chicken anti-rat for mSn were added to stain remaining surface mAb-labeled Sn. Cells were gated in a FCS/SSC dot plot to exclude cell debris and dead cells were excluded using propidium iodide labelling (PI, Sigma-Aldrich, Schnelldorf, Germany). Cells were analyzed with a FACSCalibur flow cytometer equipped with a blue (λ_ex_ = 488) and red diode lasers (λ_ex_ = 635). The used band pass filter were FL1 (515–545 nm); FL3 (670 long pass) and FL4 (653–669 nm). Mean fluorescence intensity (MFI) for surface staining was calculated relative to cells stained without primary mAbs (MFI_background_). The amount of internalization was calculated as follows: [MFI_60 min_ − MFI_background_] / [MFI_0 min_ − MFI_background_] × 100.

### 3.6. Western blot

Cells were lysed using 2% Triton X-100 in TNE buffer (25 mM Tris-HCl (Sigma-Aldrich), 150 mM NaCl (VWR, Leuven, Belgium) and 5 mM EDTA (Sigma-Aldrich, Schnelldorf, Germany)) with Complete protease inhibitor cocktail (Roche, Vilvoorde, Belgium) for 60 min at 37 °C as previously described [35]. Briefly, Laemlli buffer (Bio-Rad, Mitry-Mory, France) was added to lyse cells with or without β-mercaptoethanol (Sigma-Aldrich) and boiled. Samples were loaded on a Mini-PROTEAN TGX precast electrophoresis gel (Bio-Rad, Mitry-Mory, France). The samples were transferred onto an Immobilon-P transfer membrane (Millipore, Molsheim, France). The membrane was blocked with Tris-buffered saline-Tween 20 before adding 5 µg/mL mAb, followed by a peroxidase-conjugated anti-mouse or anti-rat secondary antibody (Dako, Glostrup, Denmark and Life Technologies, Frederick, MD, USA). Chemiluminiscence was measured after 5 min of substrate incubation with SuperSignal West Pico Chemiluminiscent Substrate (Thermo Scientific, Rockford, IL, USA) using a Genoplex Chemi camera (VWR, Leuven, Belgium).

### 3.7. Competitive binding assay

MAbs were biotinylated with EZ-link NHS-SS-biotin (Thermo Scientific, Rockford, IL, USA) following the manufacturer’s instructions. Cells were kept in a 96-well plate with V-bottom on ice during the entire experiment. 10 µg/mL unbiotinylated-mAbs were added to the cells and surface binding of mAb to Sn was allowed. After 60 min, biotinylated-mAbs were added at 5 µg/mL. Unbound mAbs were washed away before adding fluorescently-labeled streptavidin, FITC for pSn and mSn, and Alexa Fluor 647 for hSn (Life Technologies, Frederick, MD, USA). Viable cells were analyzed after PI staining with flow cytometry. MFI was calculated relative against cells incubated with phosphate-buffered saline (PBS) instead of unbiotinylated-mAbs taking background fluorescence into account.

### 3.8. Red blood cell binding assay

RBC binding assay was performed using a previously described protocol [48]. Briefly, Sn^+^-cells were treated with 10 mU/mL *Vibrio cholerae* neuraminidase (Roche, Vilvoorde, Belgium) to remove all the sialic acids. MAbs were added to the cells, after 60 min human RBC were added to allow mAbs to bind with hSn, pSn, or mSn and prevent RBC rosetting. As a positive control, no mAbs were added to the cells. Rosette formation was analyzed using light microscopy and the percentage positive cells, with at least four bound RBC, was calculated.

### 3.9. Statistical analysis

Statistical analysis was performed using a student’s *T*-test. Significant difference was shown when the p-value is lower than 0.05.

## 4. Conclusions

In this study, new anti-Sn mAbs were developed. MAbs cross-reactive with both hSn and pSn were identified, but not with mSn. The greater amino acid sequence diversity between mSn and hSn or pSn most likely accounts for this, since there is only 30% amino acid sequence identity between mSn and hSn [3]. For mSn, a new anti-mSn mAb was developed with better binding properties compared to the commonly used mAb 3D6. Our results showed that the newly developed mAbs and the commonly used mAbs, here used as a reference, recognize a different epitope. All mAbs can inhibit sialic acid-binding, which suggests that these are all reactive with the first, N-terminal variable domain of Sn. Previous research showed that, depending on the epitope recognized by a mAb, differences can be observed on the internalization of surface proteins. Here, we observed no major differences in internalization when using mAbs recognizing different epitopes, suggesting that this has no major effect on Sn internalization. Further research regarding internalization is needed for a better understanding of the internalization capacity of Sn and to allow the use of Sn-specific mAbs as a possible therapy to target Sn^+^-cells.

## Figures and Tables

**Table 1:** Overview of developed hybridoma clones. A selection was made by screening the hybridomas with enzyme-linked immunosorbent assay (ELISA) for recognition of human sialoadhesin-positive (hSn^+^), porcine sialoadhesin-positive (pSn^+^), and murine sialoadhesin-positive (mSn^+^) cells. * Indicated clones were selected for further experiments after screening with ELISA, fluorescent microscopy and flow cytometry.

Clone	Recognition			Isotype
	hSn	pSn	mSn	
10H1 *	Yes	No	No	mIgG1 kappa
12A4 *	Yes	Yes	No	mIgG1 kappa
14E5	Yes	No	No	mIgG1 kappa
17B12	Yes	No	No	mIgG2b kappa
21B7	Yes	Yes	No	mIgG1 kappa
22F3	Yes	No	No	mIgG1 kappa
24B5 *	Yes	Yes	No	mIgG1 kappa
24F6	Yes	Yes	No	mIgG1 kappa
25B7 *	Yes	Yes	No	mIgG1 kappa
25F3	Yes	Yes	No	mIgG1 kappa
26B2	Yes	Yes	No	mIgG2b kappa
26G1	Yes	No	No	mIgG1 kappa
30A6 *	Yes	Yes	No	mIgG1 kappa
30E6 *	Yes	Yes	No	mIgG1 kappa
SySy94	No	No	Yes	rIgG1 kappa
SySy304	No	No	Yes	rIgG1 kappa

**Table 2:** Clustering of the different mAb based on the site on Sn they bind. Each column indicates the mAbs that are grouped in the same cluster for, respectively, hSn, pSn, and mSn.

hSn			pSn		mSn	
Cluster 1	Cluster 2	Cluster 3	Cluster 1	Cluster 4	Cluster 5	Cluster 6
12A4	10H1	7D2	12A4	41D3	SySy94	3D6
24B5			24B5			
25B7			25B7			
30A6			30A6			
30E6			30E6			

## Supplementary Materials

The following are available online at http://www.mdpi.com/2073-4468/5/1/7.

## Abbreviations

The following abbreviations are used in this manuscript:

Ag: antigen
CHO: Chinese hamster ovary
DAPI: 4′,6-diamidino-2-phenylindole
DOAJ: Directory of open access journals
ELISA: enzyme-linked immunosorbent assay
FITC: fluorescein isothiocyanate
HIS: polyhistidine
HIV-1: human immunodeficiency virus type 1
HRP: horseradish peroxidase
hSn: human sialoadhesin
ITS-X: Insulin-Transferrin-Selenium-Ethanolamine supplement
KLH: keyhole limpet hemocyanin
LD: linear dichroism
mAb: monoclonal antibody
ME: mercaptoethanol
MDPI: Multidisciplinary Digital Publishing Institute
MFI: mean fluorescent intensity
mSn: murine sialoadhesin
PI: propidium iodide
PF: paraformaldehyde
PRRSV: porcine reproductive and respiratory syndrome virus
pSn: porcine sialoadhesin
RBC: red blood cell
SER: sheep erythrocyte receptor
Siglec: sialic acid-binding immunoglobulin-like lectins
Sn: sialoadhesin
Sn^+^: sialoadhesin positive
TLA: Three letter acronym
