# Supplementary material for: Development and Characterization of New Species Cross-Reactive Anti-Sialoadhesin Monoclonal Antibodies
Source: Antibodies (Basel). 2016 Mar 23;5(2):7. doi: 10.3390/antib5020007 (PMC6698821; doi:10.3390/antib5020007)
Supplement: Supplementary file 1 [file antibodies-05-00007-s001.pdf]

Development and Characterization of New Species Cross-Reactive Anti-Sialoadhesin Monoclonal Antibodies

Marjorie De Schryver, Hanne Van Gorp, Inge Hoebeke, Bauke De Maeyer, Karen Ooms, Isabel Pintelon, Louis J. Maes, Paul Cos, Hans J. Nauwynck and Peter L. Delputte

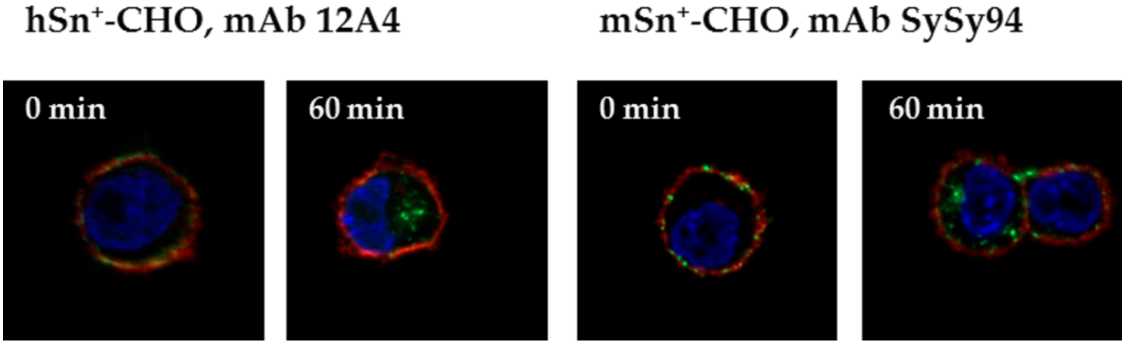

Figure S1. Internalization of Sn induced by Sn-specific mAbs. Newly developed mAbs 12A4 and SySy94 were added to the cells for 60 min at 4 °C to allow only surface staining. Cells were then fixed (0 min) or were brought to 37 °C to induce internalization (60 min). Cells were permeabilized so internalized vesicles could be observed. Secondary Alexa Fluor 488-labeled antibodies were added to the cells (green) along with Texas Red-X phalloidin (Life Technologies) to stain cortical actin (red). Nuclei were stained with 4',6-diamidino-2-phenylindole (DAPI) (blue).

© 2016 by the authors; licensee MDPI, Basel, Switzerland. This article is an open access article distributed under the terms and conditions of the Creative Commons by Attribution (CC-BY) license (http://creativecommons.org/licenses/by/4.0/).
